# Supplementary material for: Enhancement of Immune Checkpoint Inhibitor-Mediated Anti-Cancer Immunity by Intranasal Treatment of Ecklonia cava Fucoidan against Metastatic Lung Cancer
Source: Int J Mol Sci. 2021 Aug 24;22(17):9125. doi: 10.3390/ijms22179125 (PMC8431244; doi:10.3390/ijms22179125)
Supplement: Supplementary file 1 [file ijms-22-09125-s001.zip › ijms-1308662-supplementary.pdf]

Supplementary Material

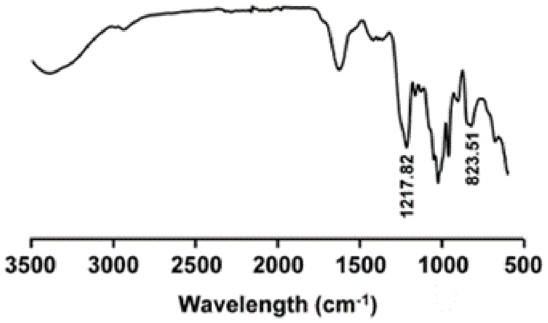

Figure S1: Fourier transform infrared spectrum of *Ecklonia cava*-extracted fucoidan (ECF).

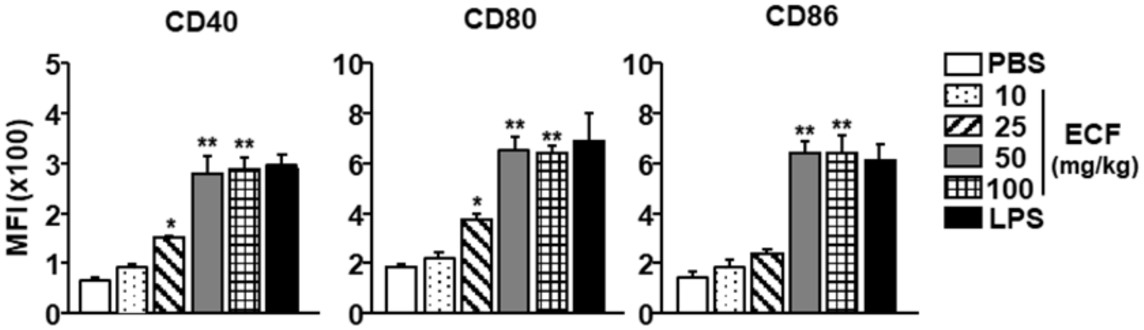

Figure S2: Dose dependent effect of ECF. C57BL/6 mice were injected intranasally with indicated dose of ECF. CD40, CD80, and CD86 expression levels in mediastinal lymph node (mLN) DCs were measured by flow cytometry 18 h after treatment (n = 6, two-way analysis of variance, $^{**}p < 0.01$).

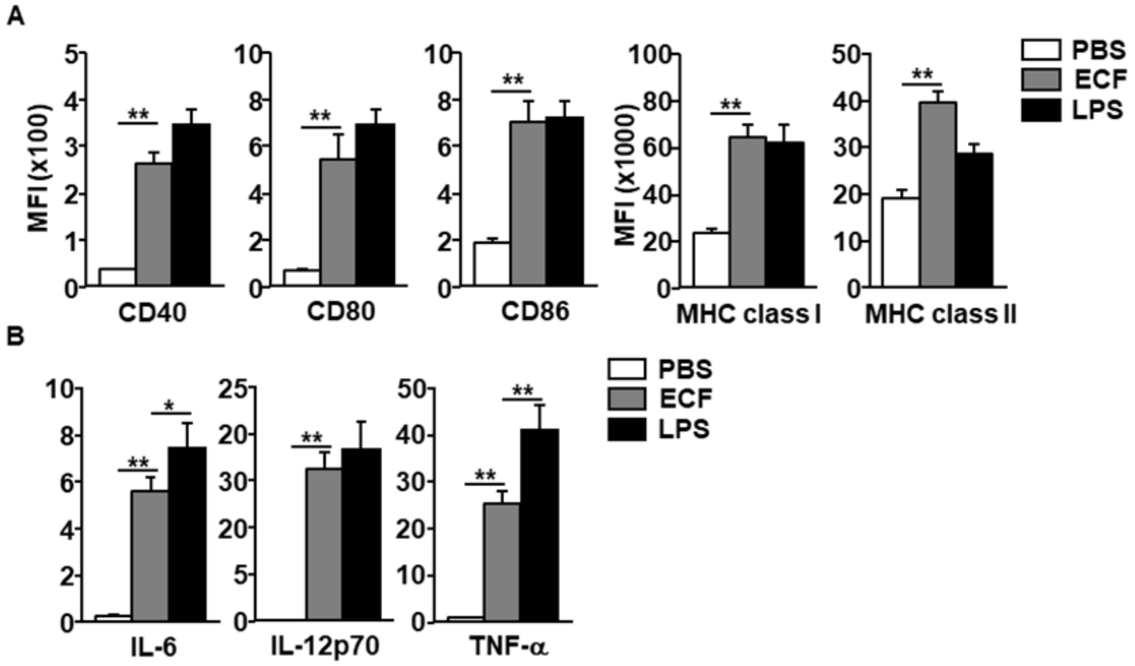

Figure S3: Intranasal injection of ECF activated mLN DCs in BALB/c mice. BALB/c mice were injected intranasally with 50 mg/kg of ECF. (A) CD40, CD80, CD86, MHC class I and II expression levels in mLN DCs were measured by flow cytometry 18 h after treatment (n = 6, two-way analysis of variance, $^{**}p < 0.01$). (B) The levels of cytokines in bronchoalveolar lavage (BAL) fluid were measured by flow cytometry 18 h after treatment (n = 6, two-way analysis of variance, $^{*}p < 0.05$ and $^{**}p < 0.01$).

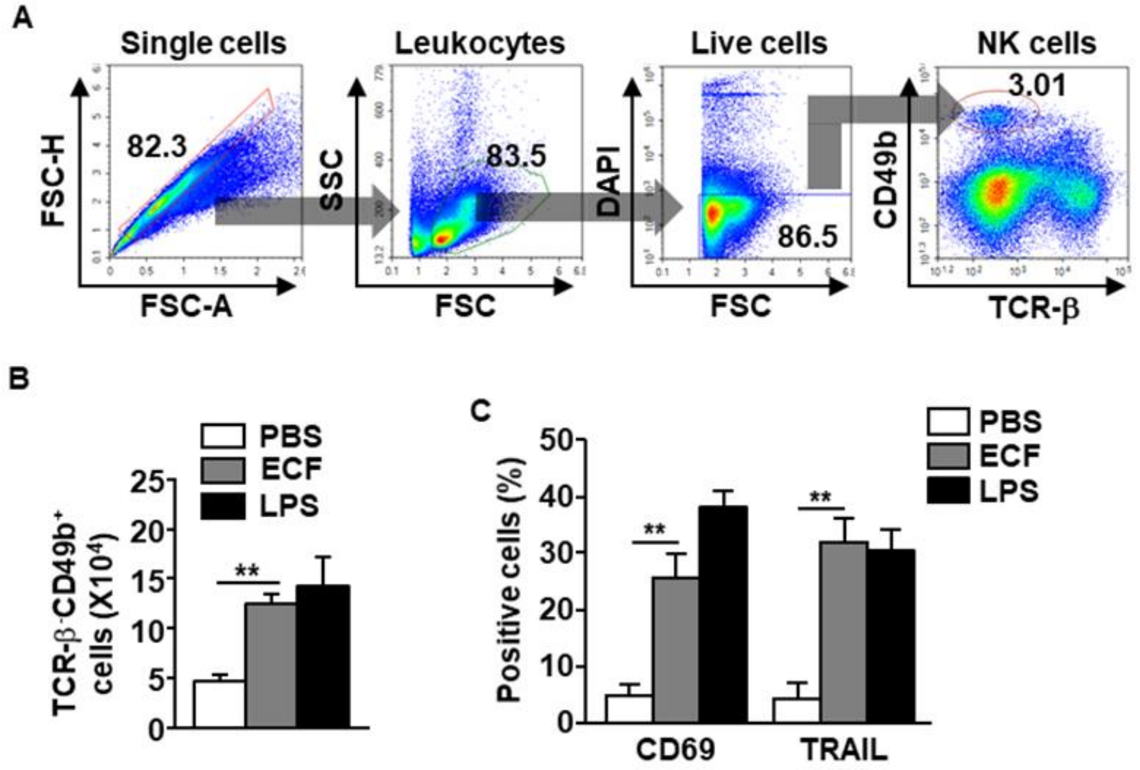

Figure S4: ECF promoted NK cell activation in BALB/c mLN. (A) Definition of NK cells in BALB/c mice are shown. (B) Average of NK cell number in the mLN (n = 6, two-way analysis of variance, **p *<0.01*). (C) CD69 and TRAIL expression levels in mLN NK cells are shown (n = 6, two-way analysis of variance, **p *<0.01*).

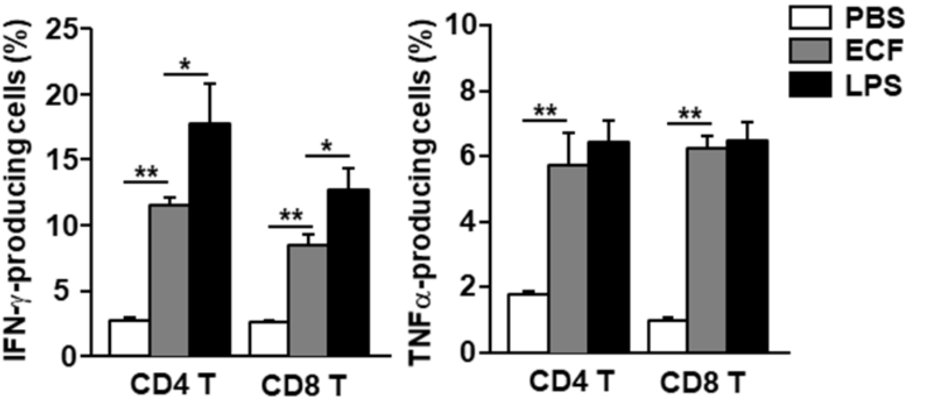

Figure S5. ECF induced Th1 and Tc1 immunity in mLN. BALB/c mice were *i.n.* administered 50 mg/kg ECF for twice 3 days interval and measured intracellular IFN-γ (left panel) and TNF-α (right panel) producing levels in CD4 and CD8 T cells (n = 6, two-way analysis of variance, *p *<0.05* and **p *<0.01*).
